# Unusual Presentation of Renal Cell Carcinoma: Gluteal Metastasis

**DOI:** 10.1155/2013/958957

**Published:** 2013-12-11

**Authors:** Yunus Emre Goger, Mehmet Mesut Piskin, Mehmet Balasar, Mehmet Kilinc

**Affiliations:** Urology Department, Meram Medical Faculty, Necmettin Erbakan University, 42080 Konya, Turkey

## Abstract

Renal cell carcinoma (RCC) has widespread and unpredictable metastatic potential. The most common sites of metastatic RCC are the lungs, lymph nodes, bones, liver, and brain; however the soft tissue metastasis is rare (2,3). Here we report a 76-year-old male patient who had renal cell carcinoma presented with gluteal metastasis. To our knowledge this is the first renal cell cancer case with gluteal metastasis at the initial diagnosis.

## 1. Introduction

Renal cell cancer (RCC) is the most frequently seen renal malignancy. Hematuria, flank pain, and the palpable mass are the classical triad of the renal cell tumor and seen only in 6–10% of the patients. The rest of the symptoms are mostly related to the paraneoplastic syndromes [1].

Approximately 20–30% of patients with localized tumours at the time of nephrectomy relapse after surgery and develop metastasis [2]. Furthermore, 25% of patients present with metastatic RCC (mRCC) at diagnosis [3].

Although renal cell carcinoma (RCC) has widespread metastatic potential striated muscle metastasis is rare and the gluteal metastasis is the one of rarest site for the renal tumor [4]. Here we report a renal cell carcinoma with gluteal metastasis as the presenting manifestation. To our knowledge this is the first renal cell cancer case with gluteal metastasis at the initial diagnosis.

## 2. Case Report

**Figure 1:**
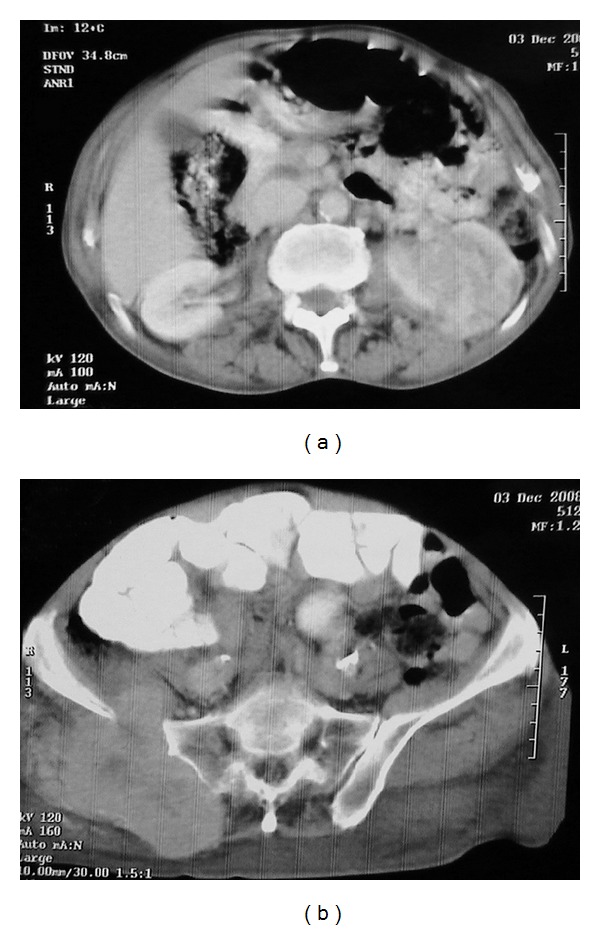
Abdominopelvic CT: (a) image showing 8 cm large mass on the left kidney and (b) CT image demonstrating 11 × 11 × 8.5 cm giant gluteal mass causing the destruction of the iliac bone and acetabulum.

**Figure 2:**
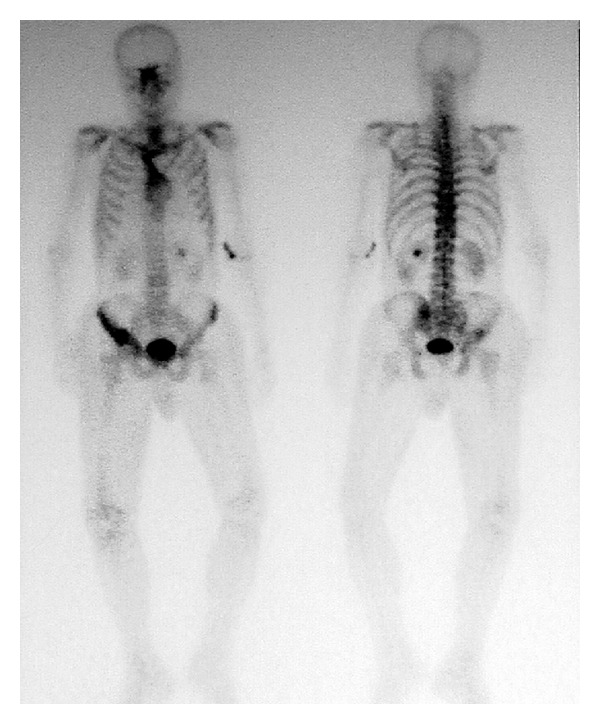
99 mTc bone scan revealed metastasis on right acetabulum and sacrum.

**Figure 3:**
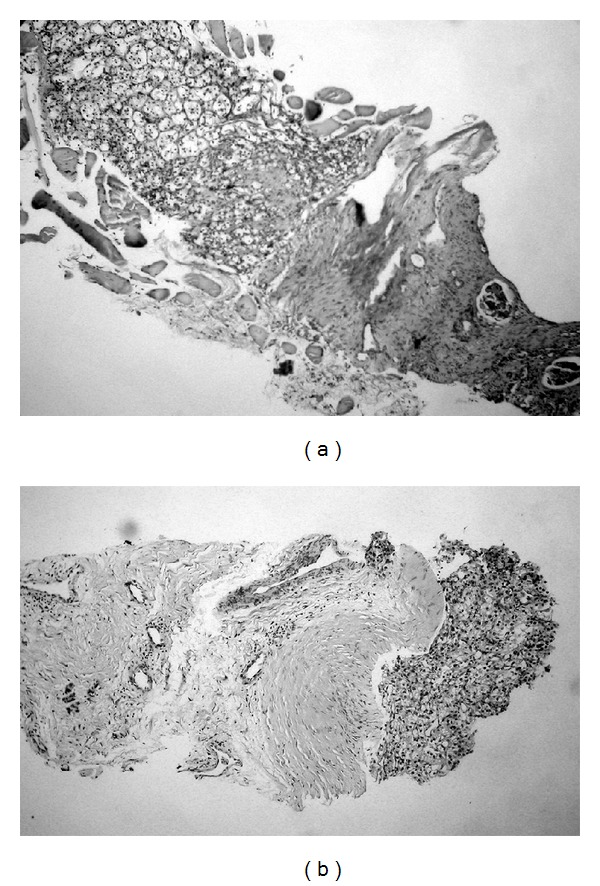
Histopathological evaluation: (a) biopsy from renal mass presenting the characteristic features of renal cell carcinoma (hematoxylin & eosin) and (b) renal cell carcinoma metastasis to gluteus muscle (hematoxylin & eosin).

A 76-year-old man presented with left flank pain and gluteal pain causing disability to walk. On physical examination there was palpable mass on right gluteal region which was noticed by the patient within last 3 months and also edema on the right leg was observed. Complete blood count revealed anaemia (haemoglobin: 10.8 g/dL). The renal function is almost good serum creatinine 0,6 mg/dL and blood urea nitrogen 49 mg/dL; performance status was poor. Abdominopelvic computerized tomography (CT) showed an 8 cm mass on lower pole of the left kidney (Figure 1(a)) and solid right gluteal mass (Figure 1(b)). There were no significant lesions on the cranial radiological evaluation but thorax CT showed some nodular lesions. 99 mTc bone scan revealed metastasis on right acetabulum and sacrum (Figure 2). RCC and the gluteal metastasis were verified with renal and gluteal biopsies performed under local anesthesia (Figures 3(a) and 3(b)). Patient died within 2 weeks.

## 3. Discussion

RCC has widespread and unpredictable metastatic potential. RCC can metastasize via venous and lymphatic routes to almost any organ; the most common metastatic sites are the lungs, lymph nodes, bones, liver, and brain [4].

In several autopsy series, about 0.4% of cases with RCC had skeletal muscle metastases [5]. However, there are few reports that show RCC metastasis to skeletal muscle in the literature [5–10]. Although the skeletal muscle has a rich blood supply, the metastases of this localization are very rare. The reasons for the rarity can be explained hypothetically as follows: (1) high pressure of tissue due to exercise-related increased blood flow preventing implantation and growth of tumor cells; (2) prevention of tumor cell growth by lactic acid production; (3) inhibition of the metastasis by skeletal muscle-derived peptidic factor; (4) protease inhibitors found in the extracellular matrix of muscle tissue might be protective factor against tumor metastasis; (5) antitumor activity of the lymphocytes and natural killers [5].

As in the present case, CT was the most commonly used radiological device in diagnosis of the muscle metastasis. MRI, fluorodeoxyglucose positron emission tomography scan, could also be used in diagnosis as an investigation tool. But biopsy is necessary to differentiate mRCC from the other skeletal muscle tumors, because primary soft-tissue tumors are more common than metastatic tumors to the skeletal muscle [7].

There are only 2 RCC reports with late gluteal metastasis following nephrectomy. All of them were on the same side with renal tumor site [9, 10]. Our case is the first one in the literature, primary RCC with bone and gluteal metastasis which was located interestingly on the opposite site of the renal tumor. This feature shows us that RCC might spread everywhere by hematogenous route.

In conclusion, according to our limited experience and on the light of the literature gluteal region is a very uncommon site for metastasis and prognosis seems poor in RCC with gluteal metastasis at the initial diagnosis.
